# The Role of Slingshot-1L (SSH1L) in the Differentiation of Human Bone Marrow Mesenchymal Stem Cells into Cardiomyocyte-Like Cells

**DOI:** 10.3390/molecules171214975

**Published:** 2012-12-17

**Authors:** Jian-Wu Zhao, Mu-Rui Zhang, Qiu-Ye Ji, Feng-Juan Xing, Ling-Jie Meng, Yan Wang

**Affiliations:** 1 Department of Orthopaedics, China-Japan Union Hospital of Jilin University, Changchun 130033, China; 2 The Key Laboratory of Pathobiology, Ministry of Education, Jilin University, Changchun 130033, China; 3 Research Center for Gene Therapy, China-Japan Union Hospital of Jilin University, Changchun 130033, China

**Keywords:** mesenchymal stem cells, cardiomyocyte-like cells, 5-azacytidine, Slingshot-1L

## Abstract

Adult cardiomyocytes (CMs) have very limited capacity to regenerate. Therefore, there is a great interest in developing strategies to treat infarcted CMs that are able to regenerate cardiac tissue and promote revascularization of infarcted zones in the heart. Recently, stem cell transplantation has been proposed to replace infarcted CMs and to restore the function of the affected tissue. This area of research has become very active in recent years due to the huge clinical need to improve the efficacy of currently available therapies. Slingshot (SSH) is a family of protein phosphatases, which can specifically dephosphorylate and reactivate cofilin and inhibit the polymerization of actin filaments and actively involved in cytoskeleton rearrangement. In this study, we found that SSH1L promoted morphology changes of microfilaments during differentiation but was inhibited by the inhibitors of actin polymerization such as cytochalasin D. Overexpression of SSH1L could promote cardiac-specific protein and genes expression. 5-Aza can induce the differentiation of hMSCs into cardiomyocyte-like cells *in vitro.* We also observed that SSH1L efficiently promotes hMSCs differentiation into cardiomyocyte-like cells through regulation and rearrangement of cytoskeleton. Our work provides evidence that supports the positive role of SSH1L in the mechanism of stem cell differentiation into cardiomyocyte-like cells.

## 1. Introduction

Myocardial infarction is a leading cause of mortality and morbidity in Western societies. Current intervention relies on the prevention of myocardial hypertrophy, fibrosis, and thrombosis [1]. After myocardial infarction, injured cardiomyocytes (CMs) in infarcted ventricular tissues are progressively replaced by fibroblasts to form scar tissues that may lead to the development of heart failure, which is a major and growing public health problem worldwide. The prognosis of heart failure is uniformly poor, despite advances in treatment [2]. Transplantation of cultured CMs into the damaged myocardium has been proposed as a method for the treatment of heart failure. Cell-based therapy has evolved quickly over the last decade in both *in vitro* and *in vivo* preclinical research and more recently in clinical trials of myocardial infarction/ischemia and heart failure [3,4,5]. Traditionally, the myocardium has been considered to have a very limited capacity for self-regeneration [6]; thus, stem cells that have the potential to differentiate into CMs may be important and powerful cellular sources to be used in these therapies [7].

Bone marrow mesenchymal stem cells (BM-MSCs) are adult stem cells that can be isolated from bone marrow aspirates. They have been expanded and differentiated into several tissue-forming cells, such as osteoblasts, chondrocytes, adipocytes, smooth muscle cells, tenocytes, myoblasts, and central nervous system cells. [8]. Mesenchymal stem cells (MSCs) can be defined as being derived from mesenchymal tissue and by their functional capacity both to self-renew and to generate a number of differentiated progeny [9]. The earliest *in vitro* demonstration that BM-MSCs can differentiate into contractile cells with a cardiac phenotype was described by Makino in 1999, in which immortalized murine MSCs were treated with 5-azacytidine (5-Aza) [10]. Tomita induced cultured adult rat BMCs into myogenic cells that express cardiac muscle cell markers, troponin 1, and β-myosin heavy chain. The induced cells were auto transplanted into myocardial scar tissue produced by a cryoinjury. The transplanted cells formed cardiac-like cells in the scar and induced angiogenesis. The heart function was improved only when the cells used in the bone marrow transplants were cultured in the presence of 5-Aza [11].

Actin filament dynamics and reorganization play a major role in cytokinesis in animal cells [12,13,14]. Cofilin and its closely related protein, actin depolymerizing factor (ADF), are key regulators of actin filament dynamics and reorganization by stimulating depolymerization and severance of actin filaments [15,16,17]. Cofilin activity is negatively regulated by phosphorylation at Ser-3 by LIM-kinase-1 (LIMK1) [18] and reactivated by theprotein phosphatase slingshot-1 (SSH1L) [19]. SSH was originally identified in Drosophila [20]. The loss of SSH function in *Drosophila* leads to disorganized epidermal cell morphogenesis, including malformation of bristles, wing hairs, and ommatidia. Thus, SSH is implicated in the formation of cellular extensions by organizing the ordered assembly of actin filaments in *Drosophila*. Phosphoinositide-3-kinase (PI3K) regulates cofilin dephosphorylation through the activation of SSH1L, and the activation of SSH1L is essential for insulin-induced membrane protrusion [21].

We have previously shown that the potential mechanism for estrogen to induce preferential osteoblastic *versus* adipocytic differentiation was through the ER-PI3K/AKT-SSH1L axis [22]. In the present study, we isolated hMSCs from bone marrow tissue, induced its differentiation into cardiomyocyte-like cells by treating with 5-Aza, and the results indicated that SSH1L promoted the differentiation of hMSCs. We hypothesized that SSH1L promoted the activation of F-actin rearrangement was critical in hMSCs differentiation into cardiomyocyte-like cells. To test this hypothesis, cardiomyocyte-like cells differentiation of SSH1L transfected hMSCs was performed in presence of inhibitors of actin polymerization such as cytochalasin D [23]. We examined that SSH1L plays an important role in hMSCs differentiation into cardiomyocyte-like cells through regulation of cytoskeleton rearrangement. Our work provides new data supporting the role of SSH1L in the mechanism of stem cell differentiation into cardiomyocyte-like cells.

## 2. Results

### 2.1. Characterization of the Isolated hMSCs

**Figure 1:**
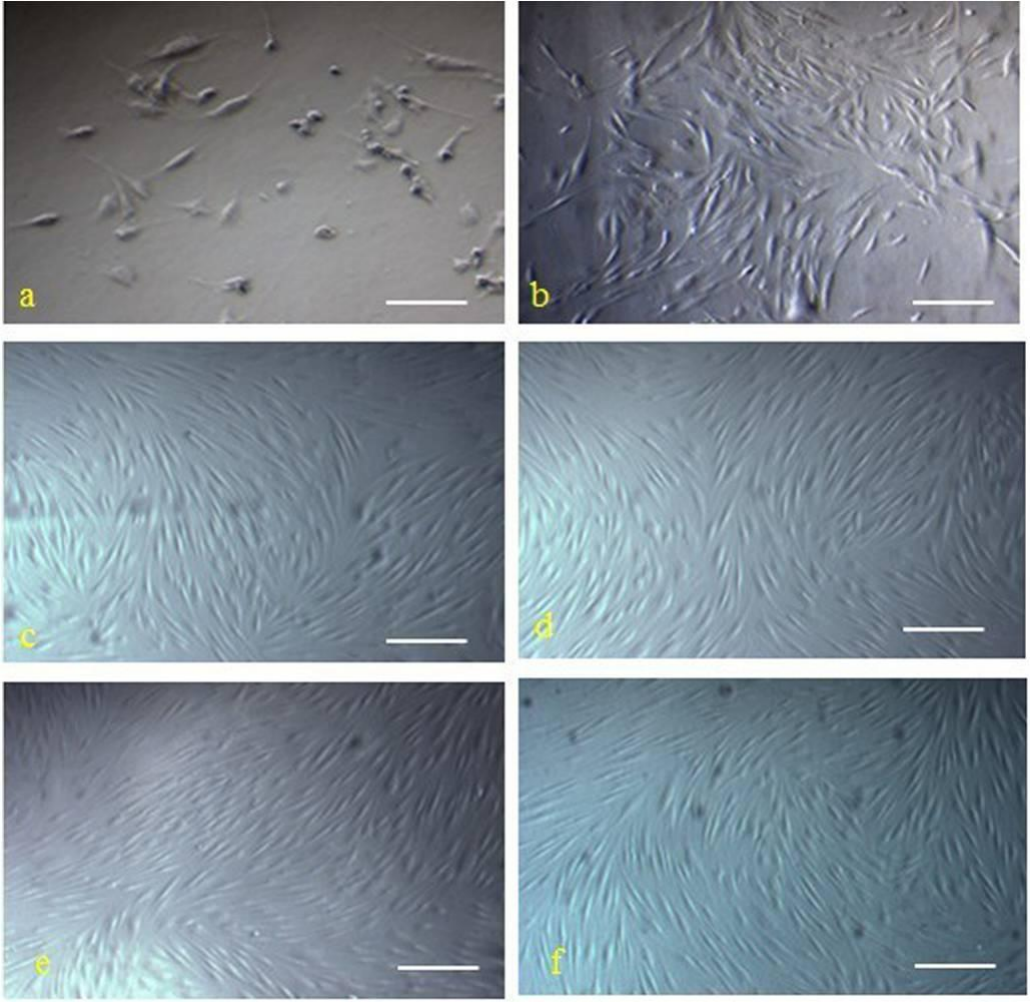
The morphology of isolated cells from bone marrow presented features of hMSCs. Cells isolated from bone marrow at 48 h after seeding (**a**, scale bar: 100 μm) and cells from individual clones grew into shoal or whirlpool shapes (**b**,scale bar: 100 μm), passage 3 (**c**), passage 6 (**d**), passage 9 (**e**), and passage 12 (**f**), respectively, were photographed under a microscope (magnification, 200×; Scale bar, 200 μm).

**Figure 2:**
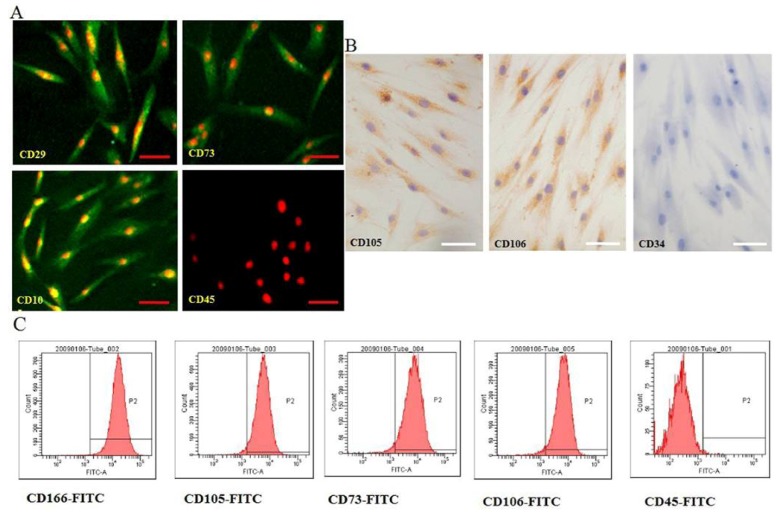
Isolated cells presented cell surface markers of the hMSCs. The expressions of indicated cell surface markers were determined on isolated hMSCs (passage 6) by immunofluorescence staining (**A**), immunocytochemical staining (**B**), and flow cytometric analysis (**C**). Images (**A**) were obtained using laser scanning confocal microscopy. Scale bar, 50 μm. Cells in the P2 gate (**C**) represented the positively stained population.

**Figure 3:**
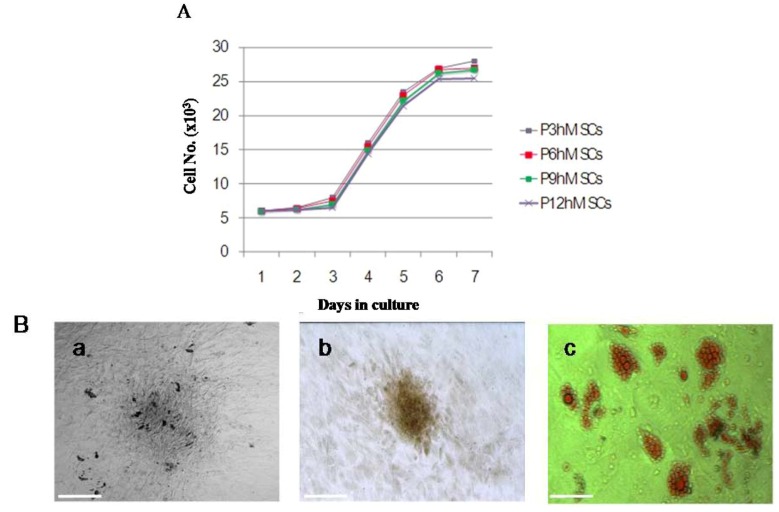
The growth cures and adipogenic, osteogenic differentiation of hMSCs. (**A**) Isolated hMSCs (8 × 10^3^) were seeded into a 24-well plate in DMEM supplemented with 10% FBS for a total of 7 days. The cell number on each day was determined with trypan blue staining; (**B**) The isolated hMSCs underwent osteogenic and adipogenic differentiation for different time periods. The cell morphology, ALP and oil red O stain were examined by differential interference contrast microscopy respectively. Scale bar, 100 μm.

The hMSCs were successful isolated from human marrow blood by density gradient centrifugation, selecting for adhering cells and sparsely distributed single adherent cells. These hMSCs were observable at 48 h after seeding, and the morphology of hMSCs was fibroblast-like and spindle-shaped (Figure 1a). The medium was changed every 3 days. At days 7 to 10, single cell-derived colonies started to form and were further cultured. By days 13 to 20, cells from the individual clones grew to approximately 90% confluence. These cells maintained their long spindle shape, attached well to the tissue culture dish, and clustered in an orderly fashion into shoal or whirlpool shapes (Figure 1b). These cell clones were passaged at a 1:3 ratio, and they preserved a fibroblast-like morphology and a constant growth rate until passage 12 (Figure 1c–f). The indicated cell surface markers of the isolated hMSCs were detected by immunofluorescence staining (Figure 2A), immunocytochemical staining (Figure 2B), and flow cytometric analysis (Figure 2C). Notably, these cells were positive for specific hMSCs markers, such as CD29, CD73, CD105, CD106, and mesenchymal cell marker CD166, but were negative for hematopoietic stem/progenitor and endothelial cell marker CD34 and white blood cell marker CD45. The growth curves of hMSCs are shown in Figure 3A. Monitoring the growth of isolated hMSCs, the growth of cells was in the latent phase for the first 2 days, entered a log phase between days 3 and 5, and then reached a plateau. The doubling time for cells in the log phase was 37 ± 1.8 h, and the cells maintained a constant growth pattern until passage 12. The hMSCs were cultured in adipogenic and osteogenic media, respectively. 11–14 days after osteogenic induction, multiple but separated nodules, formed by the clustering of cells, became visible (Figure 3B-a), which further transformed into typical bone trabeculae structures by days 22 to 24 and hMSCs began to express and became positive for ALP (Figure 3B-b). 14 days after adipogenic induction, oil red O staining of adipogenic cultures was performed. Many lipid droplets were observed in the cytoplasm of induced hMSCs (Figure 3B-c).

### 2.2. Determination of the Optimal 5-Aza Concentration to Induce Cardiomyocyte-Like Cells Differentiation of hMSCs

Different concentrations of 5-Aza (0, 1, 5, 10, 20, and 50 μM) were added to the isolated hMSCs when they were incubated in L-DMEM supplemented with 10% FBS to determine the optimal concentration of 5-Aza to induce the hMSCs to differentiate into cardiomyocyte-like cells. The evolution of the morphology was monitored, and the viability of the cells subjected to various concentrations of 5-Aza was assessed by the MTT assay.

**Figure 4:**
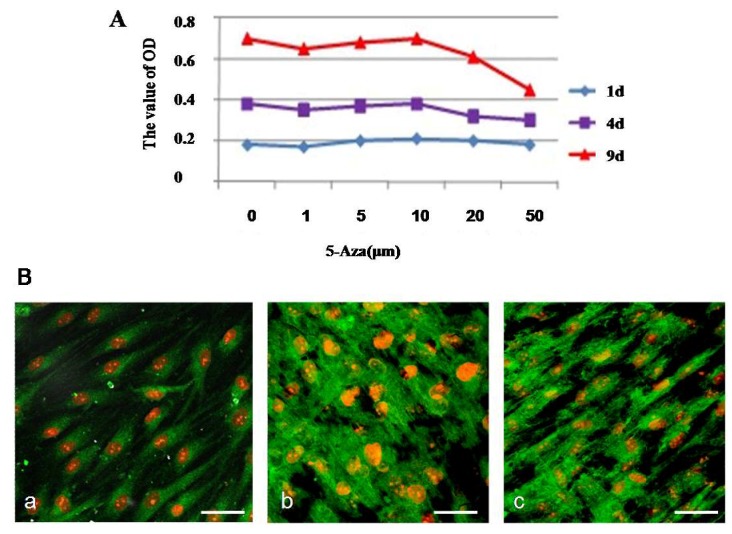
The optimal concentration of 5-Aza treatment induced cardiomyocyte-like cells differentiation. (**A**) Cells were induced with 5-Aza at different concentrations (6 replicates for each condition) for 24 h, and the medium was replaced with L-DMEM supplemented with 10% FBS. Cell proliferation was calculated at 1, 4, and 9 days after induction using the MTT assay according to the manufacturer’s protocol; (**B**) The microfilament changes of cardiomyocyte-like cells induced by 5-Aza were photographed under a microscope. The expression of microfilament was detected by immunofluorescent staining as described under the Experimental. Scale bar, 20 μm.

The treatment of hMSCs with different concentrations of 5-Aza affected the viability of hMSCs to various degrees. The levels of metabolic activity of hMSCs were assessed by the MTT assay. As shown in Figure 4A, the viability of the cells was not affected at any concentration of 5-Aza after 1 day, indicating that the cells might be in an adaptation period. When induced after 4 days, the cell activity increased consistently. When the concentration of 5-Aza was less than10 μM, the influence over the hMSCs viability was minor. But when the concentration of 5-Aza was more than 20 μM, the morphology images showed cell retraction shorter, growth condition poor, almost no proliferation and cell death constant (Figure 4B). Taking the cell viability results together with the morphology analysis of hMSCs into cardiomyocyte-like cells, we concluded that 10 μM is the most appropriate concentration of 5-Aza for induction; therefore, this concentration was used in further experiments.

### 2.3. 5-Aza Treatment Induced Cardiomyocyte-Like Cells Differentiation of hMSCs in Vitro

**Figure 5:**
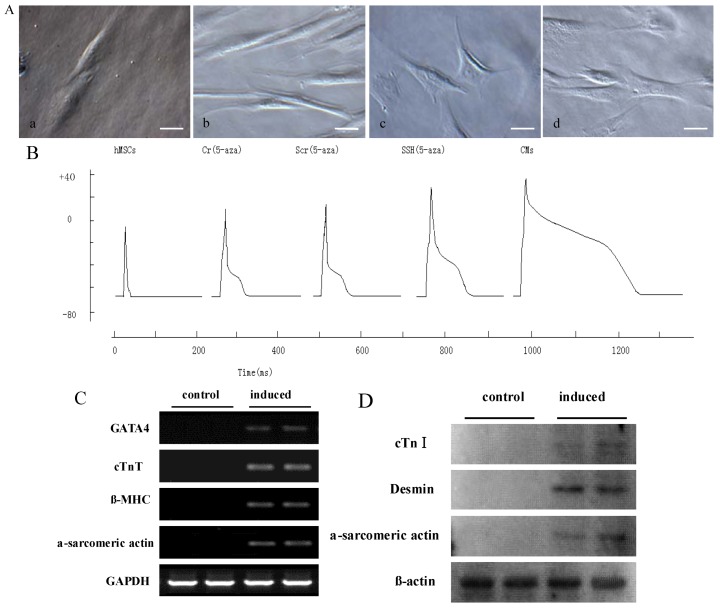
The effect of 5-Aza on cardiomyocyte-like cells differentiation of hMSCs *in vitro.* (**A**) The morphological changes of cardiomyocyte-like cells differentiation were photographed under a microscope. Scale bar, 20 μm; (**B**) Currents recorded in a patch clamped cell. As shown in Figure, compared with the hMSCs group, Cr and Scr group fired apparent action potentials. There were no significant differences on action potential between Cr and Scr groups. Compared with Cr and Scr group, SSH1L prolonged APD and elevated population spike. The voltage steps of SSH group applied in 100 mV increments from −70 mV to +30 mV from a holding potential of −70 mV. But contrast to CMs, SSH group cells have weaker action potential; RT-PCR (**C**) and Western blot (**D**) analysis for the expression of CM-specific genes and proteins, which were increased after 5-Aza treatment.

We carried out a series of detections to confirm the effect of 5-Aza on the cardiomyocyte-like cells differentiation of hMSCs. The hMSCs at the fourth passage were treated with 10 μM 5-Aza for 4 weeks to induce cardiomyocyte-like cells differentiation. After 5-Aza treatment, compared with the untreated group (Figure 5A-a), the morphology of cardiomyocyte-like cells gradually increased in size and lengthened in one direction after 1 week of induction (Figure 5A-b) and assumed a multiple branch morphology after 2–3 weeks (Figure 5A-c). The cells also connected with neighboring cells forming myotube-like structures after 3–4 weeks of induction (Figure 5A-d). After forming the whole cell configuration, the action potentials were elicited by intracellular injection of depolarizing current pulses (1 nA with 2 ms duration) at a frequency of 1 Hz. 

**Figure 6:**
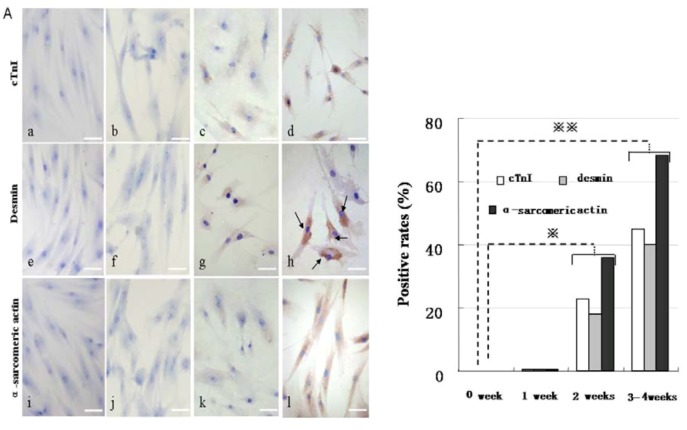
Immunocytochemical staining analysis of cardiac-specific protein expression after 5-Aza treatment. (**A**) Immunocytochemical staining of CM-specific proteins such as cTnI (upper panels), desmin(middle panels), and α-sarcomericactin(lower panels) by inverted microscopy. The positive rates were shown in (**B**). a, e, and i were controls; b, f, and j were treated with 5-Aza for 1 week; c, g, and k for 2 weeks; d, h, and l for 3–4 weeks. Scale bar, 50 µm.

The patch clamp studies were used to confirm that hMSCs induced by 5-Aza could fire action potentials. The hMSCs and rat myocardial cell were used as experimental controls. These electrophysiologic changes suggest that 5-Aza plays an important role in hMSCs differentiation into cardiomyocyte-like cells (Figure 5B). RT-PCR analysis for the expression of CM-specific genes showed that the induced cells expressed GATA4, cTnT, β-MHC, and α-sarcomeric actin; in addition, these CM-specific genes were not expressed in undifferentiated hMSCs (Figure 5C). Western blot and Immunocytochemical staining analysis both revealed that induced cells were strongly positive for CM-specific proteins such as cTnI, desmin, and α-sarcomeric actin; whereas non-induced cells were negative for them (Figure 5D, Figure 6). These results indicate that 5-Aza was successful in inducing the cardiomyocyte-like cells differentiation of hMSCs.

### 2.4. Effect of SSH1L on Cardiomyocyte-Like Cells Differentiation of hMSCs

**Figure 7:**
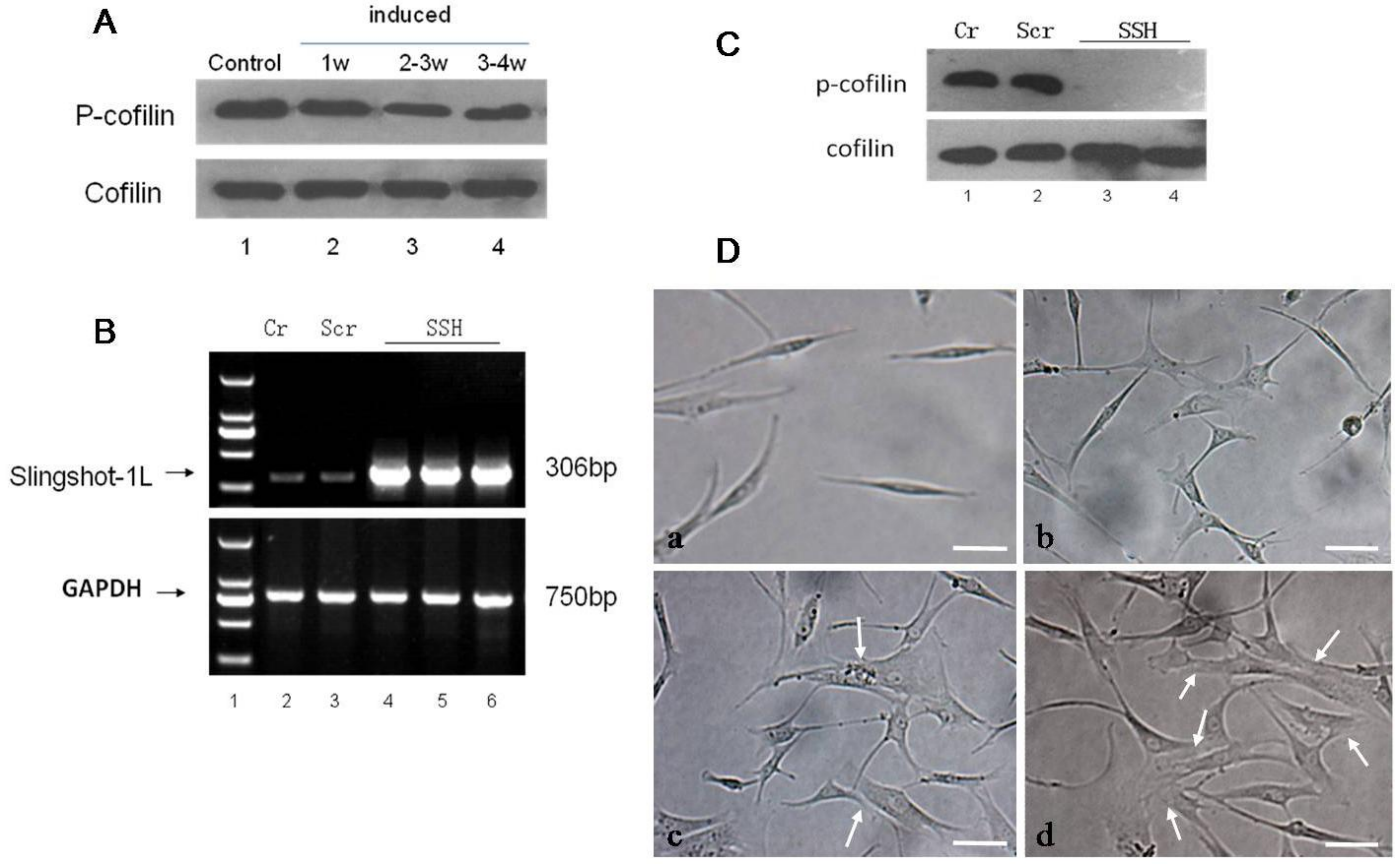
The effect of SSH1L on the morphology of cardiomyocyte-like cells differentiation of hMSCs. (**A**) We induced cardiomyocyte-like cells differentiation of hMSCs after the optimal concentration of 5-Aza treatment for 1 week, 2–3 week, 3–4 week, and then tested that the expression of endogenous protein phosphor-cofilin decreased with the induction time increasing; (**B**) The hMSCs were transfected with the retroviral vector pLNCX-SSH1L to overexpress SSH, and the empty vector transfected group (Scr) and an untransfected group (Cr) were compared as controls. In addition, the expression of SSH1L was observed; (**C**) Compared to Cr and Scr, the endogenous protein expression of phosphor-cofilin decreased apparently in the SSH group of transfectedhMSCs (SSH1L), and there are no difference between Cr and Scr; (**D**) The effect of SSH1L on the morphological changes of cardiomyocyte-like cells induced by 5-Aza were photographed under a microscope . Arrows indicate the myotube-like structures. Scale bar, 20 μm.

**Figure 8:**
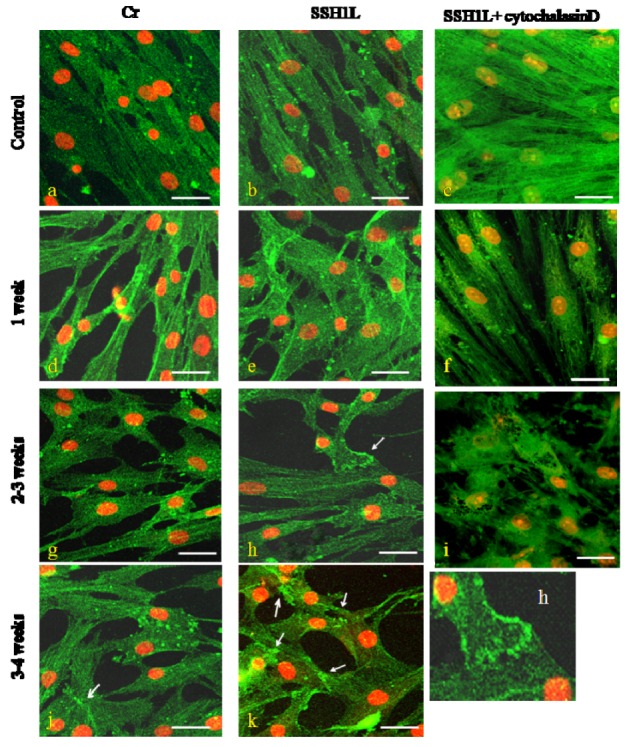
The effect of SSH1L on the changes of microfilament of cardiomyocyte-like cells differentiation induced by 5Aza. The microfilament changes of cardiomyocyte-like cells induced by 5-Aza were photographed under a microscope (bar, 20 μm). In control groups (left panels) the actin microfilaments joined one another forming myotube-like structures; and in SSH group (middle panels) showed actin microfilament junctions forming intercalated disk-like structures; but in cytochalasin D group (right panels) showed no F-actin bundles, only F-actin clumps were visible and cell death constant. microscopy. (h): Arrow indicate the intercalated disk-like structures; (j): Arrow indicate the myotube-like structures; (k): Arrow indicate the myotube-like structures or intercalated disk-like structures. The changes of microfilament were detected by immunofluorescent staining as described under the Experimental. Scale bar, 50 µm.

Our previous results suggested that the potential mechanism for estrogen to induce preferential osteoblastic *versus* adipocytic differentiation was through the ER-PI3K/AKT-SSH1L axis. We decided to research the effect of SSH1L on 5-Aza induced cardiomyocyte-like cells differentiation of hMSCs. Compared to control group, the protein expression level of endogenous phospho-cofilin decreased apparently in hMSCs induced by 5-Aza, especially 2–3 week group (Figure 7A). We successfully constructed the retroviral vector pLNCX-SSH1L which was transfected into hMSCs to overexpress SSH1L. An empty pLNCX retroviral vector transfected group (Scr) and an untransfected group (Cr) were used as experimental controls (Figure 7B). As shown in Figure 7C, SSH1L can suppress the protein expression level of phospho-cofilin, which indicated that SSH1L was activated during the differentiation process. The morphology of cardiomyocyte-like cells that were induced by 5-Aza was observed (Figure 7D). When compared with the Cr group and the Scr group (Figure 7D-a), the SSH group of transfected hMSCs showed multiple branches already after one week (Figure 7D-b) and connecting with adjoining cells and forming myotube-like structures after 2–3 weeks (Figure 7D-c).The myotube-like structures further increased after 3–4 weeks of culture (Figure 7D-d). The image of Figure 5B suggested that the action potential of SSH group was stronger than Scr and Cr group. These results indicated that the transfected hMSCs overexpressing SSH1L are more effective in differentiating into cardiomyocyte-like cells than the two control groups. In addition, we detected microfilament changes during hMSCs differentiation into cardiomyocyte-like cells induced by 5-Aza. Without induction, the hMSCs microfilaments of the Cr and SSH groups were all long straight fiber bundles along the cell’s major axis with regular arrangement (Figure 8a,b). After induction with 5-Aza for 1week, the Cr group hMSCs microfilaments were basically along the cell’s major axis but with an irregular arrangement, while the SSH group hMSCs microfilaments spread to all directions and interweaved into the reticular structure (Figure 8d,e). After induction for 2–3 weeks, the Cr group hMSCs microfilaments spread into all directions, while the SSH group hMSCs microfilaments joined one another and the actin gathered together forming intercalated disk-like structures (Figure 8g,h). After induction for 3–4 weeks, the Cr group hMSCs microfilaments joined one another forming myotube-like structures, while the SSH group hMSCs actin gathered together in microfilament junctions and formed intercalated disk-like or myotube-like structures (Figure 8j,k).

**Figure 9:**
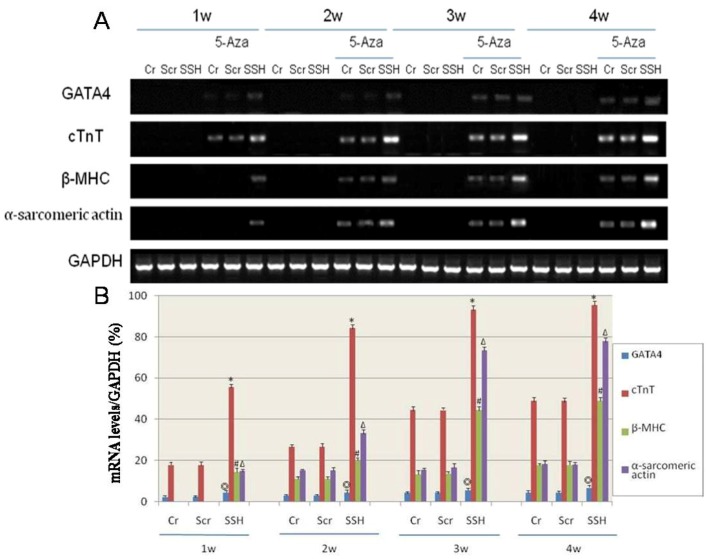
The effect of SSH1L on the expression of cardiac-specific genes of cardiomyocyte-like cells differentiation induced by 5-Aza. Cardiac-specific genes were detected by RT-PCR (**A**). (**B**): The qualified data image of cardiac-specific genes expression (*p* < 0.05 GATA4:◎; cTnT:*; β-MHC:#; α-sarcomericactin:∆). The cardiac-specific genes increased along with the increased induction time, and the expression level was much higher in the SSH group than in the other two control groups.

The SSH group hMSCs preincubated with cytochalasin D showed no F-actin bundles, only F-actin clumps were visible (1, 2 weeks); cell retraction shorter, growth condition poor, almost no proliferation and cell death constant (2 weeks) (Figure 8c,f,i). RT-PCR analysis of mRNA expression for cardiac-specific genes in hMSCs showed that the genes were not detected in the Cr, Scr, and SSH groups of hMSCs without being induced by 5-Aza. After being induced by 5-Aza for 1 week, hMSCs expressed GATA4 and cTnT, which are early phase makers of the cardiomyogenic lineage. The SSH group of transfected hMSCs began to express β-MHC and α-sarcomeric actin, which are late markers of cardiac lineage, already after 1 week. After being induced by 5-Aza for 2 weeks, the Cr and Scr group of hMSCs also express β-MHC and α-sarcomeric actin. The mRNA expression level for cardiac-specific genes increased along the time of induction, with the cardiac-specific gene expression levels being much higher in the SSH group of hMSCs than in the other two control groups (Figure 9).

**Figure 10:**
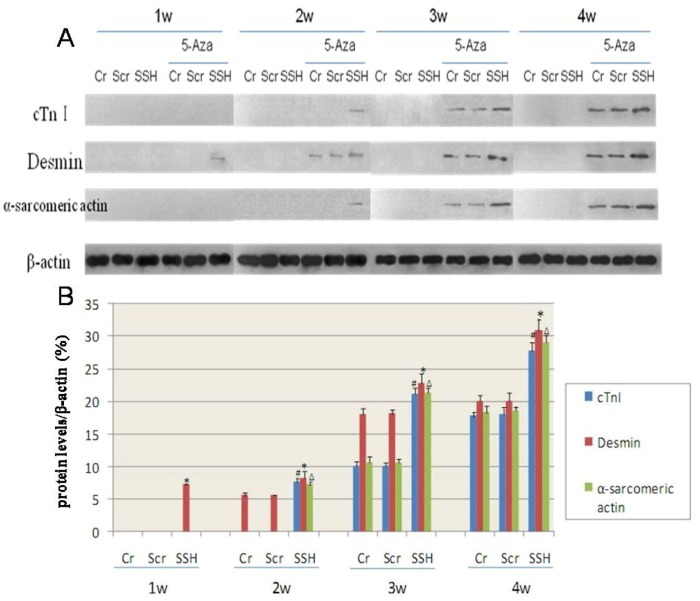
The effect of SSH1L on the expression of cardiac-specific proteins of cardiomyocyte-like cells differentiation induced by 5-Aza. Cardiac-specific proteins were detected by western-blot (**A**). (**B**): The qualified data image of cardiac-specific proteins expression (*p* < 0.05 cTnI:#; Desmin:*; α-sarcomericactin:∆). The cardiac-specific proteins increased along with the increased induction time, and the expression level was much higher in the SSH group than in the other two control groups.

Western blot analysis showed that the expression of cardiac-specific proteins were not detected in the Cr, Scr, and SSH groups of hMSCs without induction by 5-Aza. After being induced with 5-Aza for 1week, the SSH group of hMSCs expressed desmin, which is known to be an early marker of myogenic differentiation. After induction for 2 weeks, both the Cr and Scr groups also showed expression of desmin; and the SSH group showed significant expression of the cardiac-specific proteins cTnI and α-sarcomeric actin. The Cr and Scr groups showed only expression of cTnI and α-sarcomeric actin after being induced for 3 weeks. The cardiac-specific protein expression increased along the period of induction, but the SSH group showed protein expression levels much higher than those of the other two control groups (Figure 10). Based on these results, we concluded that SSH1L plays an important role in stimulating early hMSCs differentiation into cardiomyocyte-like cells through regulating cytoskeleton rearrangement.

## 3. Discussion

Cardiovascular diseases have become a leading threat to healthcare systems throughout the world. Since CMs have a very low self-regenerative capacity, the transplantation of cultured CMs into the myocardium has been proposed as a new therapy for the treatment of heart failure. A recent study revealed that transplanted fetal CMs could survive in heart scar tissue [24]. It has been demonstrated that 5-Aza in culture medium induces BM-MSCs to differentiate into the myogenic lineage [5,25]. In agreement with these reports, we have established a cardiomyogenic cell line (CMG) from human MSCs that can be induced to differentiate into cardiomyocyte-like cells *in vitro* by culturing these cells in medium supplemented with 5-Aza. When cultured in the presence of 5-Aza, the cells expressed a number of CM-specific genes including GATA4, cTnT, β-myosin heavy chain, and α-sarcomeric actin. The expression of troponin, desmin, and α-sarcomeric actin were also detectable at the protein levels. However, the mechanisms involved in the differentiation of hMSCs into cardiomyocyte-like cells are still not known in detail. As previously reported, the cytoskeleton can play an important role in the process of hMSCs differentiation. It is known that the SSH family contains SSH1L, SSH2L, and SSH3L in mammals, and they can specifically dephosphorylate and reactivate inactive Ser-3-phosphorylated cofilin (p-cofilin) [26]. Cofilin nucleates actin polymerization by severing actin filaments to generate free barbed ends and also increases the rate of actin depolymerization, thus maintaining a pool of actin monomers [27,28]. It has been demonstrated that mechanical stimulation can influence cytoskeletal patterns and encourage hMSCs differentiation to specific lineages [29]. We hypothesized that there is an interaction between cytoskeleton rearrangement and the process of cardiomyocyte-like cells differentiation. Furthermore, we hypothesized that SSH1L may elicit an important outcome in the process of hMSCs differentiation. 

In this study, the retroviral vector pLNCX-SSH1L was transfected into hMSCs to enhance the level of endogenous SSH1L expression and to evaluate its effect on cardiomyocyte-like cells differentiation. We designed the experiment with a control group of hMSCs that were not transfected (Cr), the SSH study group (hMSCs that were transfected with a viral vector encoding SSH1L) and the actin polymerization inhibitor group. The three groups were induced with 5-Aza and the evolution of the morphology was analyzed. In control groups, the actin microfilaments joined one another forming myotube-like structures, while the SSH group showed actin microfilament junctions forming intercalated disk-like structures, but the cytochalasinD group showed no F-actin bundles, only F-actin clumps were visible and cell retraction shorter, growth condition poor, almost no proliferation and cell death constant. On the basis of these experiments, we drew the conclusion that SSH1L plays an important role in hMSCs differentiation into cardiomyocyte-like cells through regulating cytoskeleton rearrangement. RT-PCR analysis of mRNA expression for cardiac-specific genes in hMSCs showed that the genes were not detected in Cr, Scr, and SSH group hMSCs without induction by 5-Aza. After being induced by 5-Aza for 2weeks, the expression level of cardiac-specific genes and proteins increased along with the increased induction time, and the expression level was much higher in the SSH group than in the other two control groups.

## 4. Experimental

### 4.1. Isolation and in Vitro Culture of hMSCs

This study was approved by the institutional review board of Jilin University. Human bone marrow was aspirated from the iliac crest of five nonosteoporosic healthy donors with written consent obtained. The ages of the volunteers (three male and two females) ranged from 17 to 43 years (mean 31.4 ± 2.6 years). They underwent a pre-donation health check-up about five weeks prior to the harvest that included health history, physical examination and X-ray. The isolation and culture of hMSCs were both performed as previously described [30], with minor modifications. Briefly, bone marrow cells were resuspended in L-Dulbecco’s modified Eagle’s medium (L-DMEM, Gibco, Grand Island, NY, USA) containing 10% fetal bovine serum (FBS, Hyclone, USA), centrifuged briefly to remove the supernatant that contained lipids, and carefully loaded onto Percoll (density 1.073 g/mL; GE Healthcare, Piscataway, NJ, USA). After centrifugation at 900 g for 30 min with the brake off, the middle layer, containing mononuclear cells, was collected and transferred to a tissue culture dish containing L-DMEM supplemented with 10% FBS (complete growth medium). Forty-four hours later, the nonadherent cells were removed, and the adherent cells were maintained in complete growth medium, with the medium changed every 3 days. From the initially isolated cells that were sparsely distributed on the plate, single-cell clones gradually expanded, which were picked at a stage containing approximately 40 to 100 cells, transferred to a new 24-well plate (passage 1), and further passaged at a 1:3 ratio. The 5-Aza induced hMSCs were preincubated with cytochalasin D, a specific inhibitors of actin polymerization, (working concentration 250 nM; Sigma-Aldrich, St. Louis, MO, USA) for 1 h before SSH1L transfection.

### 4.2. Patch Clamp Recordings

A whole-cell patch clamp technique was used to record AP characteristic [31]. A small aliquot (0.1 mL) of suspension of the isolated cells were placed into a 1.0 ml chamber, which was mounted on an inverted microscope (IX70, Olympus, Tokyo, Japan). The pipette resistances were controlled in 2–3 MΩ when filled with the electrode internal solution. The external solution was replaced using a peristaltic pump at a speed of 2 mL/min. Only quiescent and healthy looking cell were randomly selected for patch clamp studies. Position the electrode orthogonally to the cell [32]. When the electrode is near enough to the cell so that it begins move away from the out-flowing solution, quickly reverse the pressure, applying a slight vacuum to the electrode until the cell "jumps" onto it. Immediately cease suction on the electrode and apply a holding potential of −70 mV to the inside of the electrode. Within a few seconds a gigaohm seal will form. The currents were recorded using an amplifier (Axon 700B; Molecular Devices, Sunnyvale, CA, USA), a digitizer (Digidata 1322A; Molecular Devices) and Clampex software (10.0.0.61, Molecular Devices). After gigaseal and membrane rupture, the electrode resistance in series to the cell membrane was compensated.

### 4.3. Mapping of Growth Curve and Determination of Cell Doubling Time

To map the growth curve, 8 × 10^3^ isolated hMSCs, growing in the log phase, were split into 24-well plates and cultured in L-DMEM supplemented with 10% FBS at 37 °C, 5% CO_2_ for a total of 7 days. Every 24 h, cells from three wells were stained with trypan blue and counted, and the average number was calculated. The cell doubling time was further calculated using Patterson’s formula, Td = *T*log2/log(*N*_t_/*N*_0_), where Td is the doubling time in h; *N*_t_ and *N*_0_ are the cell numbers at time t and time 0, respectively; and *T* is the time in which the cell number increased from *N*_0_ to *N*_t_.

### 4.4. Flow Cytometry Analysis

Cultured adherent hMSCs were harvested with 0.25% trypsin/ethyleneediamine tetra acetic acid, washed with phosphate-buffered saline (PBS) once, and incubated with the relevant primary antibodies (1:50 for all antibodies used; NeoMarker, Fremont, CA, USA) at room temperature for 30 min. After washing with PBS two more times, the cells were incubated with the corresponding secondary antibodies with fluorescein isothiocyanate-conjugated goat anti mouse IgG (1:50; Santa Cruz, CA, USA) at 4 °C in the dark for 30 min. Labeled cells were washed with PBS twice and analyzed by fluorescence-activated cell sorting (BD Biosciences, San Jose, CA, USA).

### 4.5. Differentiation of hMSCs

The isolated hMSCs at passage4 were seeded into a 3.5-cm tissue culture plate and cultured in L-DMEM supplemented with 10% FBS (complete hMSC growth medium) for 24 h. The cells were then washed with PBS and cultured in L-DMEM supplemented with 10% FBS and10 μM 5-Aza (Sigma), and the medium was changed every 4 days for a total induction period of 4 weeks for the cardiomyocyte-like cells differentiation. To determine the optimal 5-Aza concentration for cardiomyocyte-like cells differentiation, 5-Aza at different concentrations (1, 5, 10, 20, and 50 μM) was added to the cell culture plate during the differentiation period. The hMSCs cultured in complete hMSCs growth medium with either the basal osteoblastogenic differentiation medium (complete hMSCs growth medium with 10^−9^ M dexamethasone, 10 mM sodium β-glycerophosphate and 0.05 mM ascorbic acid) or the basal adipogenic differentiation medium (complete hMSCs growth medium with 0.01 mg/mL insulin, 0.2 mM indomethacin, 0.5 mM 3-isobutyl-1-methylxanthine and 0.2 μM dexamethasone), with the medium changed every 4 days.

### 4.6. Cell Proliferation Assay

The hMSCs were seeded into a 96-well plate and cultured in L-DMEM supplemented with 10% FBS for 24 h, and the cells were washed with PBS. Then, the medium was replaced with serum-free L-DMEM for another 5 h. The cells were further induced with 5-Aza at different concentrations (6 replicates for each condition) for 24 h, and the medium was replaced with L-DMEM supplemented with 10% FBS. Cell proliferation was calculated at 1, 4, and 9 days after induction using the MTT assay according to the manufacturer’s protocol.

### 4.7. Immunocytochemical Staining

Cells were fixed in 4% paraformaldehyde at room temperature for 20 min and stained with the indicated antibody (1:100) at room temperature for 1 h, followed by anti-rabbit secondary antibody (1:200; Wuhan Biosynthesis Boster, Wuhan, China). The signals were developed with diamino- benzidine (Wuhan Biosynthesis Boster).

### 4.8. Immunofluorescence Microscopy

Cells were grown on glass coverslips for 48 h, fixed with 4% paraformaldehyde at room temperature for 20 min, washed 3 times in PBS and permeabilized with 1% Triton X-100 for 5 min. Cells were blocked with 3% BSA and incubated with primary antibodies for 1 h. The concentrations of primary antibodies (NeoMarker) were: β-actin 1:50; anti-CD29 1:50; anti-CD73 1:50; anti-CD10 1:50; anti-CD45 1:50. After the incubation, cells were washed and treated with Alexa Fluor-labeled secondary antibodies and rinsed several times with PBS. Immunofluorescence images were examined by confocal microscopy.

### 4.9. Transfection of hMSCs

Overexpression of SSH1L in cardiomyocyte-like cells was conducted by transfection of the wild-type human SSH1L-targeting sequence (TCGTCACCCAAGAAAGATA) using a pLNCX retroviral vector. The pLNCX-SSH1L retroviral vector was constructed as described previously [20] and transfected with lipofectamine 2000 (Invitrogen, Carlsbad, CA, USA), following the manufacturer’s protocol.

### 4.10. Reverse Transcription-Polymerase Chain Reaction (RT-PCR)

Total RNA was extracted from the cells using TRIZOL reagent (Invitrogen) according to the manufacturer’s protocol. To generate cDNA, total RNA and the Revert Aid first strand cDNA synthesis kit (MBI) were used according to the manufacturer’s instructions. Next, the resultant cDNA was used for each PCR. Primers were designed and utilized for SSH1L (5'-CACAAGCATGCAGGTGATCT/TATGTGCATCCTTCCTGCTG-3'), GATA4 (5'-CTGTCATCTCACTATGGGCA/CCAAGTCCGAGCAGGAATTT-3'), cTnT (5'-AGAGCGGAAAAGTGGGAAGA/CTGGTTATCGTTGATCCTGT-3'), β-MHC (5'-CGAGGCAAGCTCACCTACAC/CATTAACAGCCTCCACGGCC-3'), and α-sarcomeric actin (5'-TCTATGAGGGCTACGCTTTG/GCCAATAGTGATGACTTGGC-3'). All the PCR products were run on agarose gel with ethidium bromide and quantified by densitometric analysis.

### 4.11. Western Blotting

Protein lysates were prepared and quantified, separated by 12% SDS polyacrylamide gels, and then transferred to polyvinylidenedifluoride membranes. The membranes were first incubated with primary antibodies against cTnI, desmin, and α-sarcomeric actin, phosphor-cofilin, cofilin and subsequently incubated with horseradish peroxidase-linked secondary antibodies. The western blot procedure was performed as previously described elsewhere [33,34,35].

### 4.12. Oil Red O Staining for Adipocytes

Cells were fixed with neutral buffered formalin at room temperature for 30 min, washed with distilled H_2_O, immersed in 70% propylene glycol briefly and stained with oil red O solution at room temperature for 10 min. Then, the cells were further dehydrated in 70% propylene glycol and observed under the microscope. To quantify the number of adipocytes, 10 random fields were photographed under 100× magnification, and the number of oil red O-positive cells was counted, with the average of 10 fields calculated and presented as a mean ± standard deviation.

### 4.13. Statistical Analysis

Quantitative data were presented as a mean ± standard deviation for at least three cell clones of hMSCs isolated independently from all five donors. Statistical significance was calculated using the Student’s t-test for paired data. A *p* value of <0.05 was considered statistically significant.

## 5. Conclusion

In short, this study systemically showed, for the first time, that 5-Aza can induce the differentiation of hMSCs into cardiomyocyte-like cells *in vitro* and confirmed that SSH1L can efficiently promote hMSCs differentiation into cardiomyocyte-like cells through regulating cytoskeleton rearrangement. Our work offers helpful theoretical support and a laboratory basis to further investigate the mechanisms of stem cell differentiation into cardiomyocyte-like cells.
